# Undiagnosed Hepatocellular Carcinoma Presenting as Nasal Metastases

**DOI:** 10.1155/2015/856134

**Published:** 2015-11-05

**Authors:** Hassen Mohammed, Rashid Sheikh, Waheed Rahman, Sally Sheta, Zeynel Dogan

**Affiliations:** Department of Otorhinolaryngology and Head & Neck Surgery, Hamad Medical Corporation, P.O. Box 3050, Doha, Qatar

## Abstract

Hepatocellular carcinoma (HCC) is a primary malignancy of the liver with up to half of cases suffering from extrahepatic metastasis in the later stages of the disease. Commonly reported and encountered metastatic sites include the lymph nodes, lung, bone, and adrenal glands. This is an effort to throw a spotlight on a rare case of metastatic HCC which presented to us as two distinct lesions in the nose. It focuses on the presentation and the steps that were taken to reach this rare and unusual diagnosis. It sparks interest from a clinical and histopathology perspective. Our cynosure is the findings of the case coupled with a probe on the possible routes of spread of HCC to sinonasal region.

## 1. Introduction

Sinonasal metastasis is extremely rare although it is reported several times in literature. However, in the case we present, the pathology was unknown and the diagnosis of a silent HCC in the liver came as a surprise to both the patient and the caregivers. Therefore, we aim to highlight the diagnostic dilemma of this case from clinical and histopathological point of view. We also hope to enlighten the readers about the approach that was taken by the histopathologist to confirm the diagnosis. We strongly felt that reporting this case with a literature review was warranted to include exploration of the existing theories of the modes of spread of HCC to the sinonasal region.

## 2. Case Presentation

**Figure 1:**
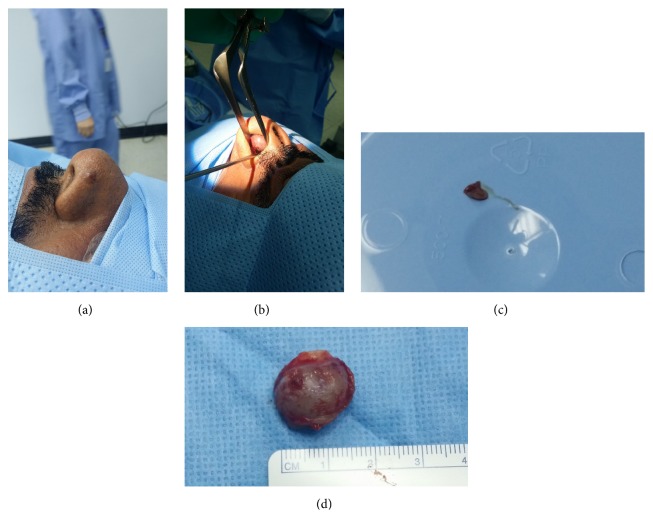
(a): Left sided raised nasal alar lesion. (b) Right sided nasal septal mass. (c) Wide local excision of left sided nasal alar lesion. (d) Excision biopsy specimen of right sided nasal septal mass.

A 58-year-old male presented to our otorhinolaryngology and head and neck surgery clinic with a recurring left sided nasal skin lesion and a right sided nasal septal mass. The two lesions were rapidly growing over the preceding 3 months and bled with minor trauma. It is pertinent to mention that the patient had no chronic illnesses and had no alarming social habits including smoking or excessive alcohol consumption. Prior to presenting at our department, the nasal skin lesion was treated multiple times with cryotherapy. On examination, the patient was conscious and alert with stable vitals and no signs of acute distress. General physical examination revealed no pallor, jaundice, cyanosis, clubbing, or edema. Cardiopulmonary examination was unremarkable. The abdomen was soft and nontender with no palpable hepatosplenomegaly or obvious stigmata of chronic liver disease. Neurological and musculoskeletal systems were intact. However, 0.5 × 0.5 cm sessile pinkish lesion (Figure 1) on the skin of the left nasal ala and 1.5 × 1.5 cm sessile pink mass (Figure 1) arising from the right nasal septum were detected on inspection of the nose and anterior rhinoscopy, respectively. Fiberoptic nasopharyngoscopy done to evaluate the posterior nose, pharynx, and supraglottic areas was unremarkable. With a preliminary diagnosis of pyogenic granuloma, and after informed consent, the patient underwent excision of both lesions. The biopsy specimens were sent for histopathological analysis (Figure 1).

**Figure 2:**
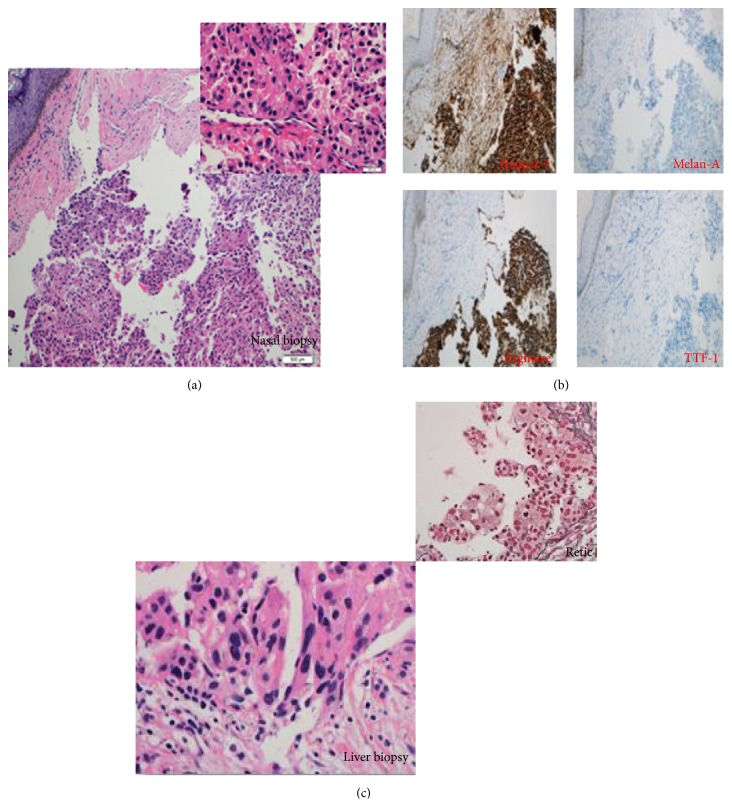
Histopathological studies. (a) H&E staining showing sheets and nests of cells divided by delicate capillaries network. Cytologically, the cells are polygonal with pink cytoplasm and markedly pleomorphic nuclei. (b) Immunohistochemistry: the tumor cells are strongly and diffusely positive with HepPar1, arginase, CD10, and pancytokeratin. Stains with TTF-1, S100, Melan-A, CK7, CK20, P63, synaptophysin, Vimentin, inhibin, PSA, RCC, CK19, and alpha-1-fetoprotein. (c) Liver biopsy reveals large atypical hepatocytes with somehow papillaroid configuration and exhibits marked nuclear pleomorphism and nuclear pseudo-inclusion.

Histopathology conducted on both the specimens showed a similar picture of malignant neoplasm composed of sheets and nest of cells divided by a delicate capillary network. Neoplastic cells were of medium-to-large size with abundant cytoplasm and fairly round uniform nuclei with prominent nucleoli. Intranuclear inclusions were also identified. Mitosis was frequently noted. The tumor had a circumscribed pushing margin covered by epidermis. Immunohistochemical study with appropriate controls was performed and showed that the neoplastic cells were positive for cytokeratin AE1/cytokeratin AE3, HepPar1, arginase, CD10 (canalicular pattern), CEA polyclonal (canalicular pattern), and CE138. The tumor was negative for TTF-1, Vimentin, CK20, CK7, p63, CK34BE12, HMB45, synaptophysin, S100, Melan-A, inhibin, PSA, CK19, alpha-1-fetoprotein, and RCC (Figure 2). Final impression was of a malignant neoplasm in both the lesions. Histomorphology and immunoprofile favored metastatic moderately differentiated hepatocellular carcinoma with a recommendation by the histopathologist to investigate the liver for a possible primary.

Based on the histopathological findings, extensive laboratory workup ensued. Complete blood count with peripheral smear, electrolytes, urine analysis, and renal function tests and lipid profile and most importantly liver function tests and serum alpha fetoprotein (AFP) were within normal limits. An abdominal ultrasound followed by MRI was requested. Results showed a cirrhotic liver with large 5 × 15 cm vascular lesion with MR characteristics suggestive of a neoplastic process, most likely hepatocellular carcinoma. The hepatic and portal vessels showed either partial or complete filling defects, all suggestive of tumor invasion. An ultrasound-guided liver biopsy revealed a diagnosis of well differentiated hepatocellular carcinoma on top of chronic hepatitis grade III/grade IV, stage III/stage IV. A whole body FDG PET scan was performed in search for distant metastasis. Multiple lung and subcutaneous nodules with moderate uptake were detected suggestive of metastasis. Interestingly, the primary HCC did not show uptake on the PET CT scan.

A multidisciplinary team plan was set for hepatic artery embolization and systemic therapy with sorafenib as well as palliative radiotherapy to the nasal area. To date, 12 months after the initial nasal lesions were excised, the patient has had no signs on recurrence of the lesions in the nose. He is being managed by the oncology and palliative care teams with regular 3-monthly imaging surveillance. A good response to therapy of both the primary and the metastatic tumors has been noted primarily because the tumors have not increased in size and no new growths have been noted. The patient clinical and general condition remain stable.

## 3. Discussion

Hepatocellular carcinoma (HCC) remains a global health concern. It is believed to be the second most common cause of cancer-related mortality worldwide after lung cancer and responsible for approximately three-quarters of a million deaths annually [1]. Cases are most prevalent in East Asia (35 per 100,000 persons per year), followed by Africa and Pacific Islands [2].

In South Asia, the rate of HCC is 7.6 per 100,000 persons per year for males and 2.8 for females. Most of the patients present in their 5th and 6th decade. Hepatitis B virus (HBV) is the main causative agent for HCC in many Asian Pacific countries although in South Asia hepatitis C virus (HCV) is the definitive contributor, seen in 60–70% of all cases. This can be attributed to the prevalence of HCV in the population, with 4.8% of general population positive for anti-HCV antibody which is closely followed by the HBV surface antigen at 2.5%. The high prevalence of HCC is also attributed to high contamination rates with aflatoxin, a toxin produced by* Aspergillus* growing on crops, which is amongst the most carcinogenic substances known [3]. In Middle Eastern countries, liver cancer is a major concern among men, especially in certain countries such as Egypt and Saudi Arabia, and to a lesser extent in other affluent countries of this region. Recent reports demonstrate that the incidence of HCC has increased sharply in the last decade [4]. It is possible that the high influx of immigrants from South Asia to Middle Eastern countries may be a significant contributor to the rising incidence and prevalence.

Approximately 50% of HCC patients will suffer from extrahepatic metastasis, the most frequent sites being the lung, regional lymph nodes, adrenal glands, and bone [5]. Nasal metastasis is extremely rare, as we found only two cases of metastatic HCC to nasal septum in published literature [5, 6]. Metastasis to the sinonasal region is usually associated with advanced disease and early mortality. The mean survival of patients ranges from 1 month to 26 months after the identification of sinonasal metastasis [7]. In our case the patient has remained alive 12 months after presentation with the nasal mass with palliative radiochemotherapy alone.

The pathogenesis of distant metastasis of HCC to the head and neck region remains obscure but one proposed route is the spread via the hepatic veins to caval venous system through the pulmonary circulation and then into arterial vessels feeding the head and neck. Another explanation is by retrograde spread via the prevertebral and vertebral venous plexus. Increases in intra-abdominal pressure can squeeze the tumor cells into the prevertebral and vertebral venous plexus, which communicate at the foramen magnum with the venous systems of the skull base as the cavernous sinus and pterygoid plexus and eventually into sinonasal region [5, 7].

For management of sinonasal metastatic lesions, various modalities have been reported. They include palliative radiotherapy, systemic chemotherapy, and transcatheter embolization to control nasal bleeding. Surgical intervention of metastatic septal lesion has been also reported as a simple modality to control local symptoms [7].

## 4. Conclusion

This report aims to sensitize the otorhinolaryngologist and head and neck surgeon and histopathologist to keep metastatic HCC on their differential list while investigating and managing such a case. HCC has the ability to spread to any region of the body without prior warning of a liver disease or pathology. The histopathologist requires a high index of suspicion to diagnose a case of HCC with no supporting clinical information.
